# Effects of Ultramarathon Running on Mitochondrial Function of Platelets and Oxidative Stress Parameters: A Pilot Study

**DOI:** 10.3389/fphys.2021.632664

**Published:** 2021-01-28

**Authors:** Florian Hoppel, Elisa Calabria, Dominik H. Pesta, Wilhelm Kantner-Rumplmair, Erich Gnaiger, Martin Burtscher

**Affiliations:** ^1^Oroboros Instruments, Innsbruck, Austria; ^2^Department of Sport Science, University of Innsbruck, Innsbruck, Austria; ^3^Department of Neurosciences, Biomedicine and Movement Sciences, University of Verona, Verona, Italy; ^4^Institute for Clinical Diabetology, Leibniz Center for Diabetes Research at Heinrich-Heine University Düsseldorf, German Diabetes Center, Düsseldorf, Germany; ^5^German Center for Diabetes Research (DZD e.V.), München-Neuherberg, Germany; ^6^Institute of Aerospace Medicine, German Aerospace Center (DLR), Cologne, Germany; ^7^Department of Psychosomatic Pain Ambulance, University Hospital for Medical Psychology and Psychotherapy, Innsbruck, Austria; ^8^D. Swarovski Research Laboratory, Department of Visceral, Transplant Thoracic Surgery, Medical University Innsbruck, Innsbruck, Austria

**Keywords:** mitochondrial function, platelets, ultramarathon running, acute kidney injury, muscle damage

## Abstract

Only a few studies have evaluated changes in mitochondrial function and oxidative stress associated with ultramarathon running. Invasive biopsies are needed to assess mitochondrial function of skeletal muscle, which may not be well tolerated by some individuals. Platelets (PLTs) as a metabolically highly active and homogenous cell population were suggested as a potentially valuable surrogate to investigate mitochondrial function. Thus, this study was aimed to evaluate mitochondrial function of PLTs and its association with individual race performance and markers of oxidative stress, muscle damage and renal dysfunction. Race performance and mitochondrial function (high-resolution respirometry, HRR) of PLTs using different substrates inducing ROUTINE, LEAK, N-pathway control state (Complex I linked oxidative phosphorylation; CI, OXPHOS), NS-pathway control state (CI + II linked OXPHOS and electron transfer pathway; ET), S-pathway control state (CII linked ET) as well as parameters of oxidative stress and antioxidant capacity, and markers of muscle and renal injury were assessed in eight male ultramarathon runners (26–45 years) before, immediately after and 24 h after an ultramarathon race (PRE, POST, and REC). Ultramarathon running induced an increase in LEAK O_2_ flux of PLT mitochondria and slight, largely non-significant changes in the oxidant/antioxidant balance. Levels of creatine kinase (CK), lactate dehydrogenase (LDH), blood urea nitrogen, and creatinine were all significantly elevated POST and remained high in REC. There were inverse correlations between race time and N-linked substrate state PRE-POST, and changes in CK and LDH levels were significantly related to PLT mitochondrial LEAK and N-linked respiration PRE. Although race-related changes in respirometry parameters of PLT mitochondria were rather small, a somewhat more pronounced increase in the relative N-linked respiration in faster runners might suggest PLT CI as indicator of physical fitness. The higher PLT LEAK PRE and diminished increase of CK during the race may represent a prophylactic preconditioning and the slight but non-significant elevation of the antioxidant potential post-race as a protective consequence of the race-related oxidative stress and potential threat to the kidney. Our findings point toward an interrelationship between mitochondrial function of PLTs, individual fitness levels and extreme physical and metal stresses, which stimulates further research.

## Introduction

Long-distance endurance competitions are physically and mentally challenging events affecting various physiological systems (Knechtle and Nikolaidis, 2018). In contrast to a standard marathon, (patho) physiological responses to an ultramarathon (any race > 42.2 km in distance) have not been evaluated extensively (Knechtle and Nikolaidis, 2018; Hoppel et al., 2019). Whereas markers of inflammation, muscle tissue damage, liver and renal function are among the most characterized (Jastrzębski et al., 2015; Knechtle and Nikolaidis, 2018; Hoppel et al., 2019), only a few studies have assessed mitochondrial function and oxidative stress associated with ultramarathon running (Knez et al., 2006; Fernström et al., 2007; Hattori et al., 2009; Sahlin et al., 2010; de Lucas et al., 2014; Mrakic-Sposta et al., 2015). Skeletal muscle mitochondria are key for cellular energy homeostasis and adaptation to physical activity (Lee and Shao, 2014; Porter et al., 2015; Christensen et al., 2016), but biopsies are needed to assess mitochondrial function, which is an invasive procedure and may not be well tolerated by some individuals. Therefore, platelets as a metabolically highly active and homogenous cell population have been suggested as a potentially valuable surrogate to investigate mitochondrial function (Kramer et al., 2014; Melchinger et al., 2019).

PLTs have been suggested to reflect the systemic effects of exercise on multi-organ systems based on existing data on the release of different cytokines, ions, metabolites and tricarboxylic acid (TCA) cycle intermediates from contracting muscles to the blood stream (primarily span-2 metabolites: succinate, malate, and fumarate), which are further affecting succinate receptor 1 (SUCNR1) positive cells such as PLTs in a dose-dependent manner (Gibala et al., 1998; Lewis et al., 2010; Högberg et al., 2011; Ariza et al., 2012; Knechtle and Nikolaidis, 2018). Moreover, recent studies have demonstrated parallels between oxidative respiration of skeletal muscle mitochondria and those of PLTs (Tyrrell et al., 2016; Braganza et al., 2019; Rose et al., 2019). Thus, the well accepted association between cardiorespiratory fitness and OXPHOS capacity of skeletal muscles (Walsh et al., 2001; Daussin et al., 2008; Pesta et al., 2011; Jacobs and Lundby, 2013; Christensen et al., 2016) might also apply (at least partly) to PLT mitochondrial function (Tyrrell et al., 2016). To the best of our knowledge, only one study has dealt with effects ultra-endurance exercise on PLT metabolism (de Lucas et al., 2014).

Of course, PLT metabolism is highly involved in a variety of systemic processes such as hemostasis, immunoregulation, inflammation and oxidative stress (de Lucas et al., 2014; Posthuma et al., 2015; Taeb et al., 2017; Melchinger et al., 2019). All of them are known to be affected by prolonged (intensive) exercise (Knechtle and Nikolaidis, 2018; Hoppel et al., 2019) and may also be involved in (muscle) tissue damage (Mastaloudis et al., 2006) and/or dysfunction of various organs (Ratliff et al., 2016). With regard to ultra-endurance exercise, increased PLT activity are of interest because it may provoke thromboembolic events under certain conditions (Posthuma et al., 2015; Taeb et al., 2017).

The mitochondrial respiratory chain represents the main source of oxygen-derived free radicals (ROS) in addition to other intracellular sources (e.g., xanthine oxidase, NADPH oxidase, peroxisomal oxidative enzymes) (Mastaloudis et al., 2004; Sahlin et al., 2010). Oxidant species may activate signaling pathways promoting cellular adaptations to exercise but may also become detrimental in certain cases (Hattori et al., 2009; Mrakic-Sposta et al., 2015; He et al., 2016). ROS rapidly react with cellular components and, following exhaustive exercise, a significant increase in ROS production can overcome antioxidant defense (resulting in oxidative stress) (Mrakic-Sposta et al., 2015; He et al., 2016). Adverse consequences may include damage of lipids, proteins and nucleic acids, perturbed cellular mechanisms or inflammation, potentially aggravated with aging, and pre-existing various diseases (Kohen and Nyska, 2002; Hattori et al., 2009; Nikolaidis et al., 2012; Mrakic-Sposta et al., 2015; He et al., 2016). An increased production of ROS as a consequence of high energy demand during exhaustive exercise like ultramarathon running is a well-established finding (Niemann et al., 2002; Knez et al., 2006; Fernström et al., 2007; Hattori et al., 2009; de Lucas et al., 2014; Mrakic-Sposta et al., 2015; He et al., 2016). This has been demonstrated in skeletal muscle mitochondria (Fernström et al., 2007) but fluctuating ROS generation during varying stress conditions may also play a key role in PLTs (Yamagishi et al., 2001; Lopez and Rosado, 2007; Melchinger et al., 2019).

Muscle damage and renal dysfunction are main adverse consequences of extreme endurance performance such as ultramarathon running and both are closely associated with inflammation (Pedersen and Hoffman-Goetz, 2000; Jastrzębski et al., 2015; Knechtle and Nikolaidis, 2018; Hoppel et al., 2019) and oxidative stress (Meyer et al., 2006; Ma et al., 2016). Based on the growing interest for a putative role of PLTs in sensing metabolic and inflammatory stresses, the primary goal of the present study was the evaluation of alterations in mitochondrial function of PLTs by ultramarathon running (Millet and Millet, 2012) and their association with performance, muscle and kidney injury. We hypothesized that there are significant interrelationships between changes in mitochondrial function of PLTs, individual race performance and markers of oxidative stress, muscle damage and renal dysfunction.

## Materials and Methods

### Study Participants

Local runners in the age group of 20–50 were selected from the entry list of all male starters. Since measurements were performed on 3 consecutive days, it was only feasible for locals to participate. The 32 eligible individuals were contacted 4 weeks prior to the event, informed about the procedure of measurements and asked to fill a questionnaire on anthropometric data, training and medical history, and smoking behavior if they consented study participation. Exclusion criteria were any disease associated with an increased risk due to the ultramarathon running event (assessed by an experienced physician), and very high training volumes with the intention to recruit only recreational participants (a recreational athlete was defined as training ≤ 15 h/week). Ten race participants (training volume: 4–15 h, median: 8.5 h) were included after providing written informed consent (Table 1). Due to the hot weather conditions[average ambient air temperature: 20.1°C (17–37°C), relative humidity: 54% (45–61%)], two participants did not complete the race and thus 8 individuals were included into the final analysis. The study was approved by the local ethics review board (University of Innsbruck, Institute of Sport Science).

**TABLE 1:** Participants anthropometric characteristics.

	***N* = 8**			
	**Median**	**Min**	**Max**	**IQR**
Age (years)	41.5	26	45	13.5
Height (cm)	180	173	191	11
Body mass (kg)	74.5	67.0	90.0	15.3
BMI (kg/m^2^)	24.0	20.7	28.1	4.2
Resting heart beat (bpm)	68	38	75	20.8
Training/week (h)	8.5	4	15	5.3

*Drop-outs not included.*

## Competition Profile and Determination of Race Time

**FIGURE 1:**
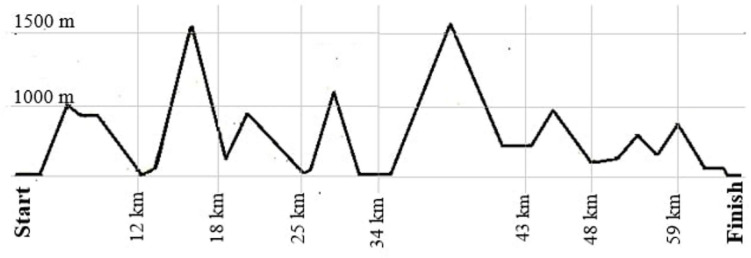
Race profile of the course.

The competition was performed as a non-stop mountain ultramarathon (67 km; approximately 4,500 m of total ascent), taking place in Gmunden, Upper Austria, Austria. Starting time was chosen individually by the competitors anytime between 3:00 – 5:00 am, cut off for finishing the race was set at 9:00 pm at the same day. Figure 1 provides a course outline of the race. Participants were wearing a heart rate monitor during competition with a recording interval of one second (Suunto Ambit 3; Suunto, Vantaa, Finland) to monitor race times, and average and maximal heart rates during the race (Suunto Movescount). Race time and average running speed were calculated using exact start and finishing times, provided by the data service of the race.

### Blood Sampling and Analyses

**FIGURE 2:**
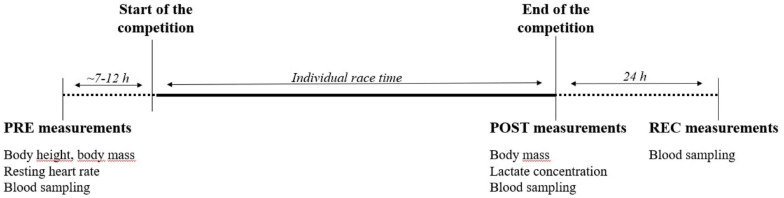
Timeline of measurements.

Venous blood sampling was performed on three consecutive days: the day before the competition (baseline condition, PRE), within 10 min after finishing the race (POST) and 24 h after the individual finish (REC) using different blood collection tubes (27 mL K3 EDTA, 6 mL Lithium Heparin, 3 mL clot activator for serum analysis; BD Vacutainer, BD diagnostics, NJ, United States). Height and resting heart rate measurements were performed PRE, body mass was determined PRE and POST. Blood lactate concentrations were only determined POST. See Figure 2 for the timeline of all measurements.

### Mitochondrial Function of Platelets

Six OROBOROS Oxygraphs 2k (Oroboros Instruments, Innsbruck, Austria) were used simultaneously to assess PLT mitochondrial function. Data were corrected for instrumental background oxygen flux and Oxygraphs were calibrated before measurement procedure every day in mitochondrial respiration media (MiR05). Each two 9 mL K3EDTA monovettes whole blood were used to extract purified PLTs for subsequent use by two specifically designed centrifugation steps. Respiratory O_2_ flux (*JO*_2_) was traced in various combinations of coupling control states and electron transfer pathways by applying a variation of the Oroboros Substrate-Uncoupler-Inhibitor Titration-8 protocol (SUIT 8) and analyzed using Dat Lab 6.0 analysis software (OROBOROS DatLab 6.0). Chemicals used and mitochondrial pathways, states and fluxes are described/defined in Tables 2, 3. The experimental procedure including sampling, sample preparation, experimental high-resolution respirometry (HRR) performance and data analysis is based on research currently published (Sjoevall et al., 2013; Fasching et al., 2016; Lemieux et al., 2017; Fasching and Gnaiger, 2018; Sumbalová et al., 2018).

**TABLE 2:** SUIT protocol, recorded respiratory states and calculated flux control ratios and flux control factors.

**Chemicals and f.c.**	**Pathways, states**	**Flux**	***FCR***	***FCF***
	ROUTINE	R	*R*	–
Pyruvate, 5 mM; Malate, 2 mM	ROUTINE (PM)	PM_*R*_	*PM*_*R*_	–
Digitonin, 200 μg/mL	N-pathway (*L*)	PM*_*L*_*	*PM*_*L*_	–
ADP, 1 Mm	N-pathway (*P*)	PM*_*P*_*	*PM*_*P*_	1-PM*_*L*_*/PM*_*P*_*
Glutamate, 5 Mm	N-pathway (*P*)	PGM*_*P*_*	*PGM*_*P*_	1-PM*_*P*_*/PGM*_*P*_*
Succinate, 10 Mm	NS-pathway (*P*)	PGMS*_*P*_*	Reference	1-PGM*_*P*_*/PGMS*_*P*_*^1^
CCCP, 0.5 μM steps	NS-pathway (*E*)	ET	*PGMS*_*E*_	1-PGMS*_*E*_*/PGMS*_*P*_*^1^
Rotenone, 2 μM	S-pathway (*E*)	S	*S_*E*_^1^*	1-S*_*E*_*/PGMS*_*P*_*
Antimycin A, 1.25 μM	ROX	–	–	–

**FCR*, flux control ratios, calculated by dividing the respective O_2_ flux by PGMS*_*P*_*. *FCF*, flux control factors, calculated by the respective flux by the next higher flux during experimental procedure. ^1^not further considered in statistics through equality to another *FCR* or *FCF*. ROX-corrected data were used for calculations of *FCR* and *FCF*.*

**TABLE 3:** Mitochondrial pathways, states, flux control ratios, and flux control factors.

ROUTINE	Respiration of intact cells measured in the physiological coupling state dependent on endogenous substrates
ROUTINE (PM)	Respiration of intact cells measured in the physiological coupling state after titration of pyruvate and malate
N-pathway	NADH electron transfer-pathway state, obtained by addition of various combinations of NADH-linked (CI-linked) substrates (pyruvate, malate, glutamate)
S-pathway	Succinate-linked (CII-linked) electron transfer-pathway state
NS-pathway	CI&CII linked electron transfer-pathway state
ROX	Residual oxygen consumption, respiration due to oxidative side reactions remaining after fully inhibition of the Electron transfer pathway
*L*	LEAK state; respiration of permeabilized cells after titration of digitonin, measured in the presence of reducing substrate(s), but absence of ADP. Low *JO*_2_ hallmarked by a back-flux of cations inducing proton leak, proton slip. Uncoupled respiration
*P*	Oxidative Phosphorylation (OXPHOS); oxidation of substrates by electron transfer to oxygen, chemiosmotically coupled to the phosphorylation of ADP to ATP. High *JO*_2_ in the presence of saturating ADP and reduced substrates. Well-coupled respiration
*E*	Electron transfer pathway (ET); respiration in the presence of a protonophore. Electrons are transferred from externally supplied reduced fuel substrates to oxygen. Experimental non-coupled respiration
**Mitochondrial fluxes**	
R	*JO*_2_ of ROUTINE respiration depending on endogenous substrates
PM*_*R*_*	*JO*_2_ of ROUTINE respiration in the presence of pyruvate and malate
PM*_*L*_*	*JO*_2_ of LEAK respiration in the presence of pyruvate and malate
PM*_*P*_*	*JO*_2_ of OXPHOS in the presence of pyruvate and malate
PGM*_*P*_*	*JO*_2_ of OXPHOS in the presence of pyruvate, malate, glutamate
PGMS*_*P*_*	*JO*_2_ of OXPHOS in the presence of pyruvate, malate, glutamate, succinate
ET	*JO*_2_ of the CI&II-linked electron transfer pathway state
S	*JO*_2_ of the CII-linked electron transfer pathway state
**Flux control ratios**	
*R*	R normalized by PGMS*_*P*_*
*PM*_*R*_	PM*_*R*_* normalized by PGMS*_*P*_*
*PM*_*L*_	PM*_*L*_* normalized by PGMS*_*P*_*
*PM*_*P*_	PM*_*P*_* normalized by PGMS*_*P*_*
*PGM*_*P*_	R normalized by PGMS*_*P*_*
*PGMS*_*E*_	ET normalized by PGMS*_*P*_*
*S*_*E*_	S normalized by PGMS*_*P*_*
**Flux control factors**	
1-PM*_*L*_*/PM*_*P*_*	PM*_*L*_* normalized by PM*_*P.*_* Step analysis of ADP titration
1-PM*_*P*_*/PGM*_*P*_*	PM*_*p*_* normalized by PGM*_*P.*_* Step analysis of glutamate titration
1-PGM*_*P*_*/PGMS*_*P*_*	PGM*_*p*_* normalized by PGMS*_*P.*_* Step analysis of succinate titration
1-PGMS*_*E*_*/PGMS*_*P*_*	PGMS*_*E*_* normalized by PGMS*_*P.*_* Step analysis of CCCP titration
1-S*_*E*_*/PGMS*_*P*_*	S*_*E*_* normalized by PGMS*_*P.*_* Step analysis of Rotenone titration

For data analysis, flux control ratios (*FCR*) and flux control factors (*FCF*) were calculated from data previously corrected for residual oxygen consumption (ROX) according to Gnaiger (2014). *FCR* are obtained by normalization of O_2_ fluxes to the respective highest flux of the experiment as reference state (Gnaiger et al., 2019). *FCF* express the relative change of respiration in a defined coupling state in a step, caused by a specific metabolic control variable (titration of a single substrate), normally being the next higher flux during experimental procedure (Gnaiger et al., 2019; Gnaiger, 2020). In contrast to normalization per cell number or mitochondrial marker, results expressed as *FCR* and *FCF* are statistically more reliable (Gnaiger et al., 2019). See Table 2 for details of the performed SUIT protocol and calculated *FCR* and *FCF*. For explanation of abbreviations please see Tables 3 and 4.

**TABLE 4:** Abbreviations.

(K3)EDTA	Ethylenediaminetetraacetic acid
ADP	Adenosine di-phosphate
ANOVA	Analysis of variance
BAP	Biological antioxidant potential
BAP/d-ROMs	BAP/d-ROMs ratio
BMI	Body mass index
BUN	Blood urea nitrogen
CARR U	Carratelli units
CCCP	Carbonyl cyanide m-chloro phenyl hydrazine, protonophore used as a potent chemical uncoupler of oxidative phosphorylation
CI	Mitochondrial respiratory complex I; ubiquinone oxidoreductase
CII	Mitochondrial respiratory complex II; succinate dehydrogenase
CK	Creatine kinase
CR	Serum creatine
d-ROMs	Determination (of) reactive oxygen metabolites
f.c.	Final concentration in the O2k chamber
*FCF*	Flux control factor, calculated by normalizing respiratory fluxes by the next highest flux of the experiment. *FCF*s express the control of respiration in a step.
*FCR*	Flux control ratio, calculated by normalizing respiratory fluxes by the highest flux of the experiment. *FCR*s express various respiratory states relative to a common reference state.
Fe^3+^/Fe^2+^	Ferric ion
H_2_O_2_	Hydrogen peroxide
HRR	High resolution respirometry
IQR	Interquartile range
*JO*_2_	Respiratory O_2_ flux
LDH	Lactate dehydrogenase
Max	Maximum
Min	Minimum
MiR05	Mitochondrial Respiration Medium, containing EGTA (0.5 mM), MgCl2. 6 H2O (2 mM), Lactobionic Acid (60 mM), Taurine (20 mM), KH2PO4 (10 mM), HEPES (20 mM), D-Sucrose (110 mM), BSA (1 g/l) (Fasching et al., 2016)
*N*	Sample size
NADH	Nicotinamide adenine dinucleotide
NADPH oxidase	Nicotinamide adenine dinucleotide phosphate oxidase
O_2_	Oxygen
O2k	OROBOROS Oxygraph 2k, the two-chamber modular system for high-resolution respirometry
*P*	Statistical *p*-value; <0.05 was considered being significant
PLT	Platelet
POST	Data measured immediately after competitional finishing
PRE	Data measured ∼12 h prior to the start of the competition
*R*	Spearman‘s rank correlation
REC	Data measured 24 h after individual finishing
ROS	Reactive oxygen species
SD	Standard deviation
SUCNR1	Succinate receptor 1
SUIT	Substrate-Uncoupler-Inhibitor Titration protocol
TCA cycle	Tricarboxylic acid cycle
η^2^	Eta-squared; statistical effect size

### Blood Analyses: Markers for Muscle and Kidney Injury and Oxidative Stress

Nine-mL K3 EDTA, 3 mL Lithium Heparin and 3 mL clot activator blood were analyzed on each sampling day at the local hospital laboratory (Salzkammergut-Klinikum, Gmunden, Austria) regarding indirect muscle, liver and renal damage markers (creatine kinase, CK; lactate dehydrogenase, LDH; serum creatinine, CR; blood urea nitrogen, BUN). With respect to the study protocol, blood was collected in non-fasting conditions. The method has been previously described elsewhere (Hoppel et al., 2019).

EDTA plasma collected from PLT sample preparation for HRR and plasma from heparinized blood (3 mL monovette) were frozen and stored in dry ice for subsequent analysis of plasma oxidative stress and biological antioxidative potential at the University of Innsbruck (Institute of Sports Science, University of Innsbruck, Austria). To evaluate the changes in oxidant species and non-enzymatic scavengers we used the d-ROMs and BAP tests (Diacron, Grosseto, Italy). The principle of d-ROMs is based on a catalytic oxidation of Fe^3+^ and free radicals to radical alkoxyl and radical peroxyl, oxidizing a chromogen (N, N,-Diethylparap-henylendiamin) to a reddish dye. The measured concentration of hydroperoxides is correlated with intensity of the dye and expressed as Carratelli units (CARR U). Reference values for a normal level of reactive oxygen metabolites are in the range of 250–300 CARR U, consistent with 20 to 24 mg/dl H_2_O_2_. BAP test is based on the reduction of Fe^3+^ ions bound to chromogen Thiocyanat to Fe^2+,^ by adding a biological sample with antioxidative defense capacity. Level of discoloration of the dye correlates with antioxidative potential of the sample and is expressed as μMol/L. Reference for good antioxidative capacity is >2,000 μMol/L (Alberti et al., 2000). To express differences between antioxidant and pro-oxidant potential, the ratio of BAP to d-ROMs has been calculated (BAP/d-ROMs). Outcomes using d-ROMs and BAP tests are robust as long as samples are stored at −20°C or lower as performed in our study (Celi et al., 2010).

### Statistics

Athletes’ anthropometric characteristics and race results are given as mean ± standard deviation (SD). Individual data of HRR measurements (Supplementary Figure 1) and oxidative status parameter (Supplementary Figure 2) are shown in the supplementary material and those of indirect tissue damage parameters in Hoppel et al. (Hoppel et al., 2019).

*FCR*, *FCF* and parameters of oxidative status, indirect muscle damage and renal function were analyzed regarding changes PRE-POST-REC using repeated one-way ANOVA with *post hoc* analysis (Bonferroni correction) for normal distributed data. Wilcoxon signed-rank test and Friedman test were applied for data not meeting ANOVA test requirements. Percentage differences were calculated for PRE-POST and PRE-REC (Tables 5, 6). PRE data were subtracted from POST (ΔPRE-POST) such as from REC (ΔPRE-REC) for *FCR*, *FCF*, d-ROMS, BAP and indirect markers of muscle damage and renal function to calculate effective changes of these parameters during the race.

**TABLE 5:** Flux control ratios and flux control factors before competition (PRE), immediately after the race (POST) and 24 h after finishing (REC).

		**After**				**Change (%)**	
	**PRE**	**POST**	**REC**	***p***	**η*^2^***	**ΔPRE-POST**	**ΔPRE-REC**
**Flux control ratios**							
*R*	0.250 ± 0.061	0.291 ± 0.064	0.246 ± 0.049	0.226	0.257	+16.34	−1.84
*PM*_*R*_	0.327 ± 0.054	0.334 ± 0.067	0.334 ± 0.030	0.854	0.031	+2.07	+2.18
*PM*_*L*_	0.199 ± 0.029	0.256 ± 0.040	0.223 ± 0.047	0.036	0.566	+28.37*	+11.82
*PM*_*P*_	0.452 ± 0.051	0.484 ± 0.058	0.454 ± 0.087	0.789	0.046	+7.01	+0.43
*PGM*_*P*_	0.566 ± 0.053	0.611 ± 0.048	0.563 ± 0.080	0.384	0.174	+7.93	−0.46
**Flux control factors**							
1-PM*_*L*_*/PM*_*P*_*	0.556 ± 0.069	0.450 ± 0.095	0.501 ± 0.095	0.005	0.731	−19.18*	−9.91
1-PM*_*P*_*/PGM*_*P*_*	0.202 ± 0.051	0.212 ± 0.085	0.199 ± 0.062	0.835	0.035	+ 5.25	−1.25
1-S*_*E*_*/PGMS*_*P*_*	0.332 ± 0.033	0.350 ± 0.039	0.323 ± 0.055	0.846	0.041	+ 8.23	+1.85

Values are shown as means ± SD, *p* values of ANOVA and η^2^ for effect size of both flux control ratios and flux control factors PRE, POST and REC. Percental changes PRE-POST and PRE-REC are given. Drop-outs are not included in statistics. * Significant change in PRE-POST *post hoc* test, *p* = 0.05. See Supplementary Figure 1 for graphic presentation of data.

**TABLE 6:** Oxidative stress parameters and indirect markers of muscular damage and renal dysfunction before competition (PRE), immediately after the race (POST) and 24 h after finishing (REC).

		**After**				**Change (%)**	
	**PRE**	**POST**	**REC**	***p***	**η*^2^***	**ΔPRE-POST**	**ΔPRE-REC**
d-ROMs (CARR U)	252.042.1	254.936.9	280.435.5	0.021	−	+0.9	+11.3*
BAP (μMol/L)	2063.4491.2	2439.6446.3	2060255.9	0.032	0.387	+18.2	−0.1
BAP/d-ROMS	8.41.5	9.61.2	7.40.7	0.014	0.575	+14.5	−12.1
Creatine Kinase (U/L)	185.3138.5	2572.91073.5	2865.4904.5	0.002	−	+1289*	+1447*
LDH (U/L)	200.024.54	373.848.70	312.274.79	<0.001	0.846	+86.9*	+56.1*
BUN (mg/dL)	14.94.6	29.16.8	25.54.4	<0.001	0.882	+95.8*	+71.4*
Creatinine (mg/dL)	0.900.05	1.540.29	1.030.14	<0.001	0.756	+71.9*	+14.7*

Values are shown as means ± SD, *p* values of ANOVA, η*^2^* for effect size and percental changes PRE-POST and PRE-REC. Drop-outs are not included in statistics. *Significant change in PRE-POST or PRE-REC *post hoc* test, *p* = 0.05. See Supplementary Figure 2 for graphic presentation of oxidative stress markers (d-ROMs, BAP and BAP/d-ROMs) and Hoppel et al for those of indirect damage markers (Hoppel et al., 2019). Ultramarathon running and platelet metabolism. Ultramarathon running and platelet metabolism.

Interactions between HRR data (Δ*FCR* and Δ*FCF* PRE-POST), athletes anthropometry and race performance parameters [age, race time, training/week, body mass index (BMI)] were analyzed by a two-tailed Spearman’s rank correlation (*r*). *R* was also used to determine interactions between PLT HRR and markers of oxidative stress, tissue damage and renal function. This was done for PRE, POST and ΔPRE-POST data. All statistics were performed using IBM SPSS Statistics 24.0 (SPSS Inc., Chicago, IL, United States). Based on data of de Lucas et al., a power of 80% (regarding changes of PLT mitochondrial function) is expected even with our eight participants (alpha set at 0.05). An effect size (η^2^) of <0.1 was considered as small effect, 0.1–0.6 as medium effect and >0.6 as large effect (Cohen, 1988).

## Results

Selected participant characteristics and race performance data are shown in Table 7. Most drop-outs during the race, including the two of the present study, occurred due to hot weather conditions (up to 37°C; average relative humidity 54%) (Hoppel et al., 2019). No serious health consequences occurred during or after the race in study participants, despite significant changes in leucocyte counts and indirect markers of potential muscular and renal damage (Hoppel et al., 2019). Race time was only correlated with BMI of the athletes (*r* = 0.862), while age, training volume and body mass change during the race (−0.2%) was not associated with running performance (Hoppel et al., 2019).

**TABLE 7:** Age and race performance of study participants compared to all male finishers.

	**Study participants (*N* = 8)**				**Race participants (*N* = 162)**			
	**Median**	**Min**	**Max**	**IQR**	**Median**	**Min**	**Max**	**IQR**
BMI (kg/m^2^)	24.0	20.7	28.1	4.2				
Training/week (h)	8.5	4	15	5.3				
Age (years)	41.5	26	45	13.5	42.0	17	75	17.0
Race time (h)	13.33	10.37	16.05	2.92	13.58	8.82	17.47	3.55
Speed (km/h)	5.1	4.2	6.5	1.2	5.0	3.6	7.8	1.5

*Two of 10 study participants (20%) and 55 of 210 race participants (26.2%) did not finish the race; drop-outs not included in the shown data.*

### Platelet Mitochondrial Function and Race Performance

**FIGURE 3:**
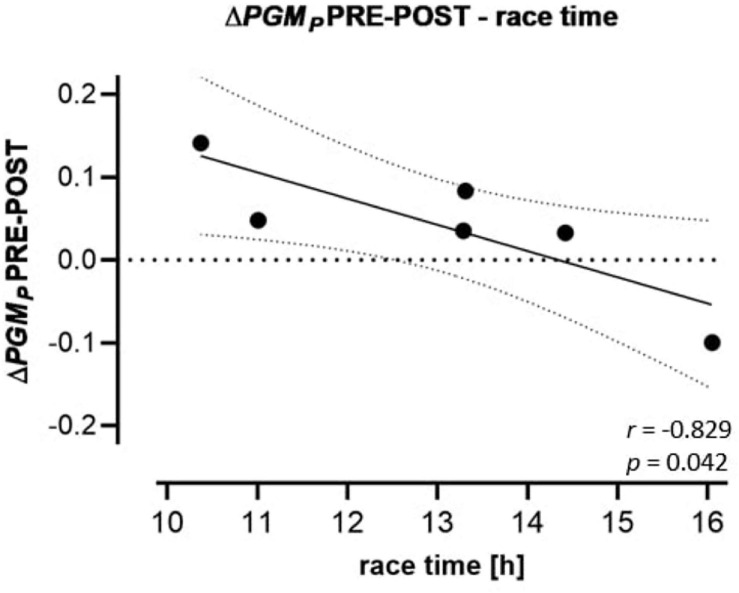
Regression (95% confidence intervals) of Δ*PGM*_*P*_ PRE-POST to race performance. Changes of FCRs of the CI OXPHOS state (substrates: PGM) PRE-POST to race time of the participants.

Calculated *FCR* and *FCF* PRE, POST and REC are presented in Table 5. Only *PM*_*L*_ (+28.4%) and 1-PM*_*L*_*/PM*_*P*_* (−19.2%) changed significantly from PRE to POST. Δ*PGM*_*P*_ PRE-POST was inversely correlated to race time (*r* = −0.829) but positively to BMI PRE (*r* = 0.826) (Figure 3).

### Blood Parameters, Oxidative Stress and Antioxidative Capacity

**FIGURE 4:**
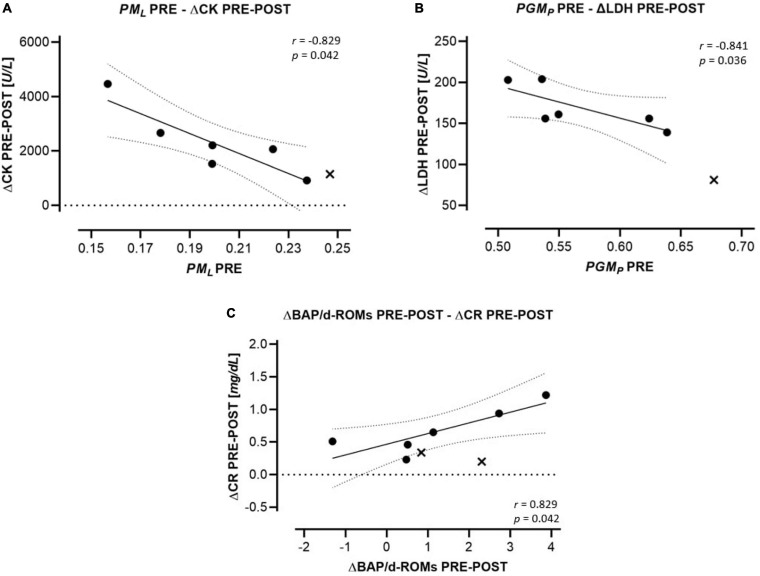
Regression (95% confidence intervals) of HRR parameters to changes in indirect markers for tissue damage. **(A)** *FCRs* of the LEAK state (*PM*_*L*_) PRE to changes of CK PRE-POST. **(B)** *FCRs* of the CI OXPHOS state (substrates: PGM) to changes of LDH (ΔLDH) PRE-POST. **(C)** Changes of oxidative stress markers (ΔBAP/d-ROMs) PRE-POST to changes of CR (ΔCR) PRE-POST. Drop-outs are marked as cross and are not included in statistics.

Markers of oxidative stress and those of potential muscular damage and renal injury are presented in Table 6. CK, LDH, BUN, and CR increased significantly from PRE to POST and remained elevated from PRE to REC. BAP/d-ROMS decreased significantly PRE-REC. Correlations between HRR parameters and markers of oxidative stress, muscle and kidney injury are presented in Figure 4. *PM*_*L*_ PRE was inversely correlated to ΔCK PRE-POST (*r* = −0.829) and *PGM*_*P*_ PRE to ΔLDH PRE-POST (*r* = −0.841). In addition, ΔBAP/d-ROMs PRE-POST was positively related to ΔCR PRE-POST (*r* = 0.829).

## Discussion

Our study shows that ultramarathon running induces an increase in LEAK O_2_ flux of PLT mitochondria and slight, largely non-significant changes in the oxidant/antioxidant balance. In addition, correlation analyses indicate a potential association between HRR parameters and those of skeletal muscle and kidney injury due to ultramarathon running.

Both *FCR PM_*L*_* and *FCF* 1-PM*_*L*_*/PM*_*P*_* express the relative amount of O_2_ being consumed in the LEAK state in relation to fully stimulated and physiologically coupled (PGMS*_*P*_*) or partially stimulated and coupled OXPHOS respiration (PM*_*P*_*) (Gnaiger et al., 2019; Gnaiger, 2020). Both positive Δ*PM*_*L*_ PRE-POST and negative Δ1-PM*_*L*_*/PM*_*P*_* PRE-POST are highlighting increased H^+^ leakage across the PLT mitochondrial inner membrane (intrinsic uncoupling), being associated with diminished respiratory efficiency (P»/O_2_ flux ratio) but increased formation of ROS (Brookes, 2005; Gnaiger et al., 2019). Basically, PLTs play a pivotal role in hemostasis, due to their accumulation at the site of injury. Whereas PLTs in a resting state are powered by glycolysis and oxidative phosphorylation, OXPHOS may be more important in activated platelets (Aibibula et al., 2018), favoring thrombus formation (Zharikov and Shiva, 2013). A previous study has shown that ultra-endurance performance is increasing PLT O_2_ consumption (de Lucas et al., 2014), which in turn triggers PLT aggregation (Melchinger et al., 2019), and hereby potentially elevating the risk of thromboembolic events in some athletes (Zaleski et al., 2016; Taeb et al., 2017). The increased intrinsic uncoupling shown in our study might therefore be interpreted as protection against thromboembolism formation (Fernström et al., 2007; Gnaiger et al., 2019).

In PLT OXPHOS, CI contributes to about 2/3 of total respiration (CI + II) at convergent electron input into the Q-junction, thus being a highly sensitive marker for a variety of diseases such as Parkinson’s or Huntington’s disease (Sjoevall et al., 2013; Zharikov and Shiva, 2013; Melchinger et al., 2019). In skeletal muscle, CI is a main source of superoxide anion radicals (O_2_^–^), but also known as a marker of cardiorespiratory fitness (Dröse and Brandt, 2008; Votion et al., 2012). If this significance of skeletal muscle CI also holds true in PLTs, the relative decrease of CI and/or increase of CII activity in slower (less fit) runners could be related to increased ROS formation, while faster (more fit) runners increasing their relative CI activity might be less affected by oxidative stress (Zharikov and Shiva, 2013). Thus, the observed relation between Δ*PGM*_*P*_ PRE-POST and race time may indicate that PLT activation and associated risk of thrombosis change with race performance. However, despite existing evidence of PLT mitochondrial parameters paralleling those of skeletal muscle (Tyrrell et al., 2016; Braganza et al., 2019; Rose et al., 2019), in data interpretation one should consider basal differences of PLT and muscle metabolic functional involvements (Lanza and Sreekumaran Nair, 2010; Melchinger et al., 2019). Further research is needed to strengthen these suggestions.

Systemic oxidative stress characterized by excessive ROS production and diminished antioxidative capacity is a well-known consequence of ultramarathon running (Hattori et al., 2009; de Lucas et al., 2014). Besides oxidative damage of carbohydrates, lipids and proteins, oxidative stress is associated with inflammation but also with the risk of muscular and renal injury (Ratliff et al., 2016; Liguori et al., 2018). Based on the relation between higher PLT mitochondrial LEAK PRE and less increase of CK during the race, we speculated that the larger LEAK PRE might represent a kind of prophylactic preconditioning.

High serum CR and acute kidney injury (AKI) are known adverse consequences of prolonged intense endurance exercise (Hodgson et al., 2017; Hoppel et al., 2019). Elevated ROS production is closely related to pathophysiological processes as it plays a key role in reducing the permeability of the glomerular basement and the vasoreactivity in renal vessels both reducing renal filtration rate (Ratliff et al., 2016). The kidney is normally protected by an upregulation of antioxidant activity, thus the slight (non-significant) increase of ΔBAP/d-ROMs PRE-POST may be considered a protective consequence to the race-related oxidative stress and potential threat to the kidney (Ratliff et al., 2016).

### Limitations

This study has some important limitations. Unfortunately, we did not have the possibility to freeze PLT samples for later analysis without impacting quality of experimental outcomes. Due to the necessity to use of fresh samples and two drop-outs during the race, the sample size of our study is small (*N* = 8). However, this is the first study evaluating PLT mitochondrial function together with markers of oxidative stress, muscle and kidney injury, thus likely stimulating further research. We were not able to determine PLT counts, protein assay or citrate synthase activity of subsamples to normalize respirometry data per cell concentration or per mitochondrial-specific marker. Although normalization of data per specific mitochondrial flux (*FCR*, *FCF*) is common and statistically precise, general limitations of ratios have to be considered (Gnaiger et al., 2019). Moreover, we did not evaluate consequences of changes in PLT mitochondrial function on hemostasis and a potential risk of thrombosis. We refer to the fact that data on indirect markers of potential tissue damage (CK, LDH, BUN and CR) have already been reported in our previous paper (Hoppel et al., 2019). However, as this study focused on alterations in PLT mitochondrial function, we believed it would be valuable to include those data for evaluation of possible associations.

### Conclusion

Our data reflect changes in respirometry parameters of PLT mitochondria as a consequence of ultramarathon running. A somewhat more pronounced increase in the relative CI ratio in faster runners may possibly represent PLT CI as an indicator of physical fitness. The higher PLT mitochondrial LEAK PRE and subsequent diminished increase of CK during the race might be interpreted (with caution) as a prophylactic preconditioning and the elevation of the antioxidant potential post-race as a protective consequence of the race-related oxidative stress and potential impact on the kidney. These findings point toward a potential interrelationship between mitochondrial function of PLTs, individual fitness levels and extreme physical and mental stresses. However, since our study population is too small to draw firm conclusions, our results should be merely considered as a valuable perspective for future investigations. Hence, further studies are needed to replicate results in a larger sample of (ultra)marathon runners of both sexes and more homogenous populations in age, BMI and training status to elucidate their clinical/practical implications.

## Data Availability Statement

The raw data supporting the conclusions of this article will be made available by the authors, without undue reservation.

## Ethics Statement

Ethical approval was not provided for this study on human participants because for minimal invasive blood sampling the approval by the local ethics review board (University of Innsbruck, Institute of Sport Science) was sufficient. The patients/participants provided their written informed consent to participate in this study.

## Author Contributions

FH, EC, EG, MB, and WK-R: planning of the project. FH, EC, WK-R, and DP: data acquisition. FH, MB, EC, and DP: drafting of the manuscript. EG, MB, WK-R, EC: project supervision and providing resources. All authors contributed to manuscript revision, read and approved the submitted version.

## Conflict of Interest

FH was temporarily employed during the project by the company Oroboros Instruments, Innsbruck, Austria. EG is the founder and CEO of Oroboros Instruments. The remaining authors declare that the research was conducted in the absence of any commercial or financial relationships that could be construed as a potential conflict of interest.

## Supplementary Material

Supplementary Figure 1 Individual and averaged data of *FCR* and *FCF* PRE, POST and REC. Calculated flux control ratios **(A)** and flux control factors **(B)**, given as single datapoints of each participant and in average. Drop-outs are marked as cross and are not included in statistics. ^∗^*p* of *post hoc* test < 0.05, ^x^adjusted *p* of ANOVA < 0.05.

Supplementary Figure 2 Individual data on d-ROMS, BAP, and BAP/d-ROMs before competition (PRE), immediately after the race (POST) and 24 h after finishing (REC). Changes of d-ROMs **(A)**, BAP **(B)**, and BAP/d-ROMs **(C)**. Drop-outs marked as cross. ^∗^*p* < 0.05.
