# Supplementary figures and images for: Effects of Ultramarathon Running on Mitochondrial Function of Platelets and Oxidative Stress Parameters: A Pilot Study
Source: Front Physiol. 2021 Jan 28;12:632664. doi: 10.3389/fphys.2021.632664 (PMC7935014; doi:10.3389/fphys.2021.632664)

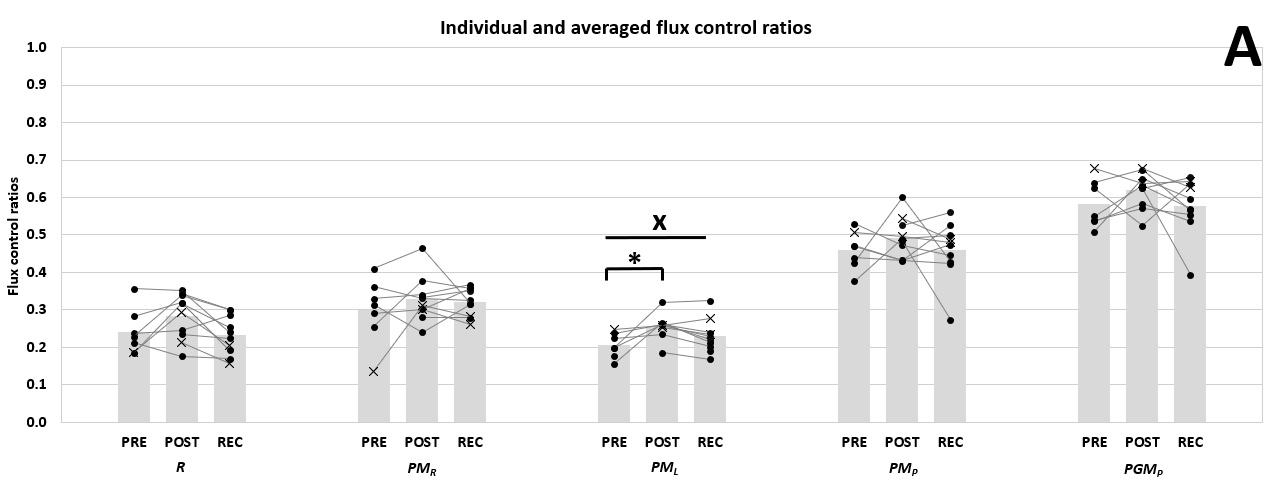

Supplement: Supplementary Figure 1 — Individual and averaged data of FCR and FCF PRE, POST and REC. Calculated flux control ratios (A) and flux control factors (B), given as single datapoints of each participant and in average. Drop-outs are marked as cross and are not included in statistics. ∗p of post hoc test < 0.05, xadjusted p of ANOVA < 0.05. [file Image_1.jpg]

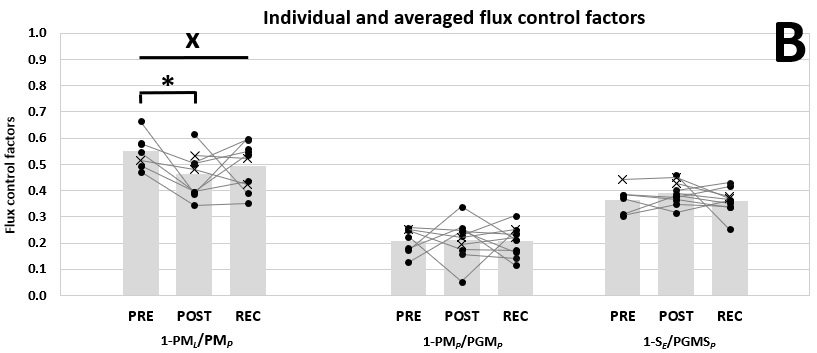

Supplement: Supplementary file 2 [file Image_2.jpeg]

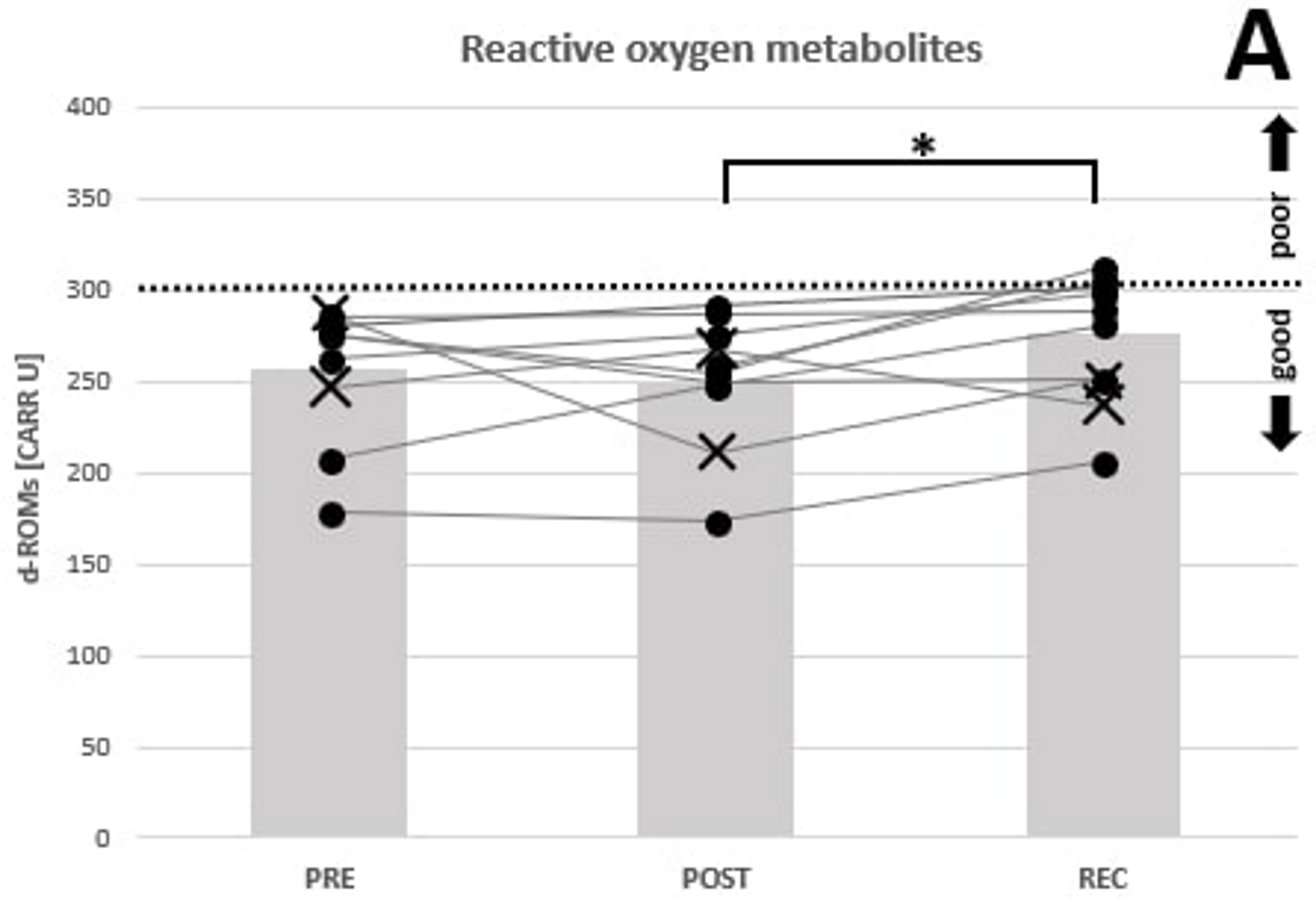

Supplement: Supplementary Figure 2 — Individual data on d-ROMS, BAP, and BAP/d-ROMs before competition (PRE), immediately after the race (POST) and 24 h after finishing (REC). Changes of d-ROMs (A), BAP (B), and BAP/d-ROMs (C). Drop-outs marked as cross. ∗p < 0.05. [file Image_3.JPEG]

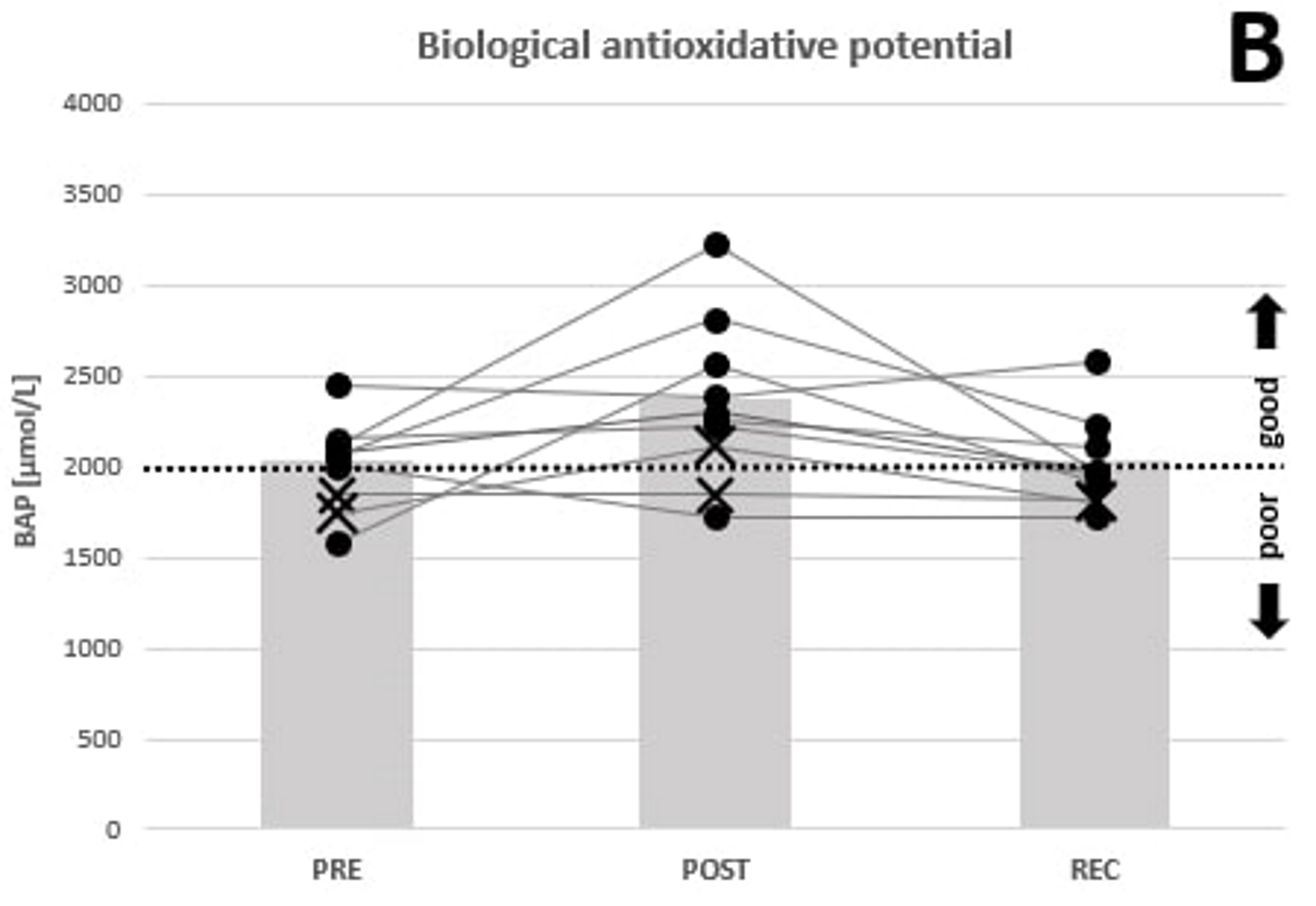

Supplement: Supplementary file 4 [file Image_4.JPEG]

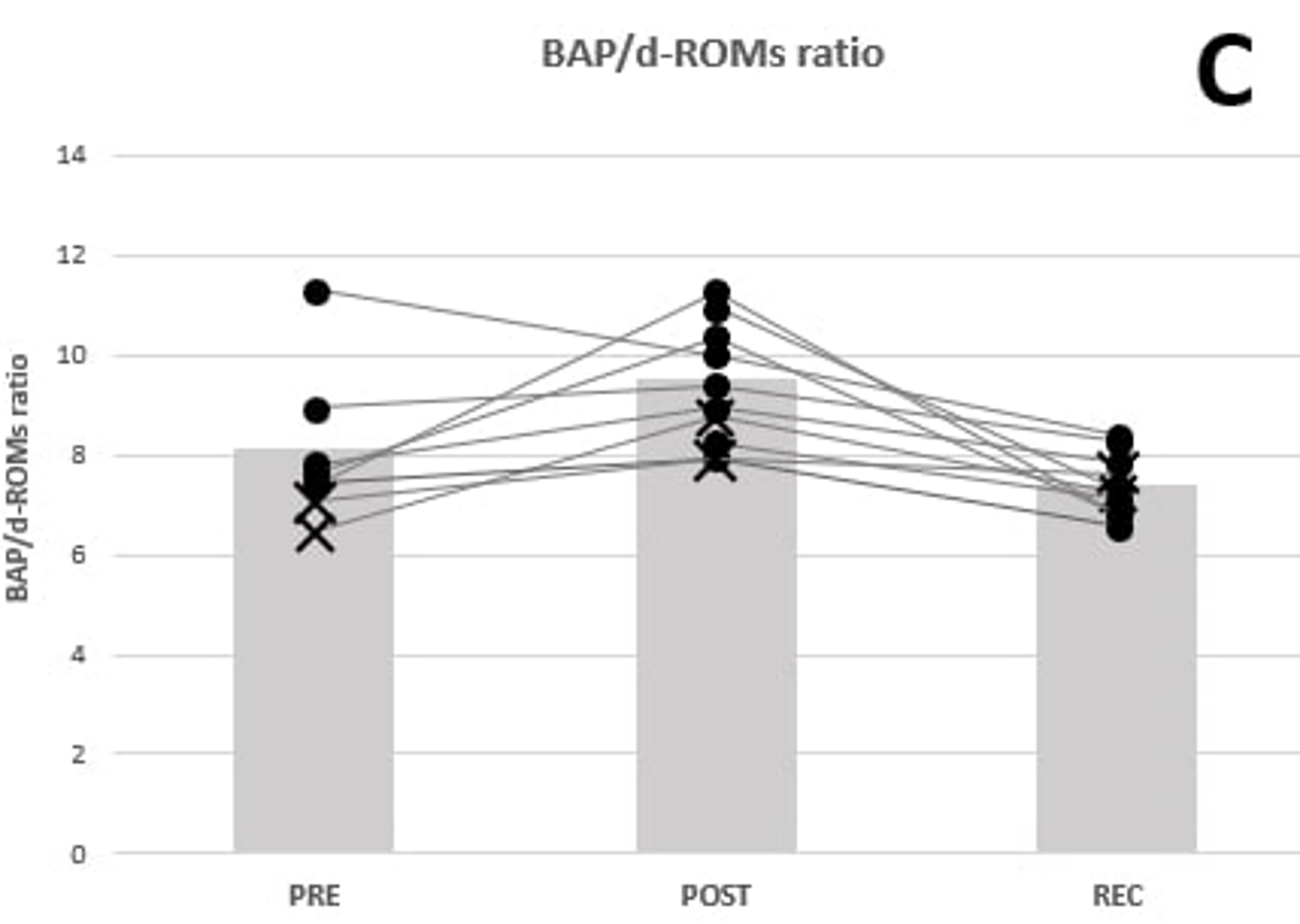

Supplement: Supplementary file 5 [file Image_5.JPEG]
